# Recent advances in understanding HIV evolution

**DOI:** 10.12688/f1000research.10876.1

**Published:** 2017-04-28

**Authors:** Sophie M. Andrews, Sarah Rowland-Jones

**Affiliations:** 1 Nuffield Department of Clinical Medicine, University of Oxford, NDMRB, Oxford, UK

**Keywords:** HIV, evolution, recombinant, mutation

## Abstract

The human immunodeficiency virus (HIV) evolves rapidly owing to the combined activity of error-prone reverse transcriptase, recombination, and short generation times, leading to extensive viral diversity both within and between hosts. This diversity is a major contributing factor in the failure of the immune system to eradicate the virus and has important implications for the development of suitable drugs and vaccines to combat infection. This review will discuss the recent technological advances that have shed light on HIV evolution and will summarise emerging concepts in this field.

## Introduction

The human immunodeficiency virus (HIV) is a major cause of morbidity and mortality worldwide, and infection leads to acquired immune deficiency syndrome (AIDS) and death in the overwhelming majority of untreated patients. Despite this, no effective vaccine or cure has yet been developed. A major obstacle in this endeavour is that rapid viral evolution essentially renders HIV a “moving target”, which also contributes to the inability of the host to control and clear the virus in natural infection. Understanding how HIV evolves is therefore of high priority, and substantial progress has been made in recent years. This review will discuss the latest technological advances that have shed light on HIV evolution and will summarise emerging concepts in the field.

## Why does HIV evolve rapidly?

HIV evolves extremely rapidly, exhibiting the highest recorded biological mutation rate currently known to science. The interpatient genome-wide nucleotide substitution rate of intracellular viral DNA may be as high as 4.1 × 10 ^-3^ (2.4 × 10 ^-3^ – 5.8 × 10 ^-3^) substitutions per site per year (s/s/y) ^1^ and, in the hypervariable regions of the envelope ( *env*) gene, could reach 5.2 × 10 ^-3^ (2.9 × 10 ^-3^ – 7.7 × 10 ^-3^) s/s/y ^2^.

The rapid rate of HIV evolution is largely attributable to the error-prone nature of reverse transcriptase, which plays an important role in viral replication yet lacks proofreading activity. This, in combination with short generation times, allows mutations to accumulate quickly within the virus at rates that differ across the genome ^3^. As the duration of infection is prolonged—with clinical latency lasting around a decade in untreated individuals—and the replicating population is large, the degree of viral diversity within a patient can be extensive ^4^.

In addition, one or more viruses may undergo recombination to produce a unique recombinant form (URF). Recombination can occur between highly divergent, closely related, or even identical viruses within a patient, and the evolutionary impact of the recombination event will be affected by the degree of divergence between the initial “parental” variants. If the resulting URF spreads amongst a population, it becomes a circulating recombinant form (CRF). Recombination of various simian immunodeficiency viruses (SIVs) is believed to have been an important contributing factor in the cross-species transmission and evolution of HIV from SIVs infecting non-human primates. It is also believed to have contributed substantially to the diversification of HIV-1 group M subtypes ^5^.

Another potential source of variation is guanosine to adenosine (G-to-A) mutation induced by the host restriction factors APOBEC3G and APOBEC3F (A3G/F) ^6, 7^, but the contribution of A3G/F to viral evolution is controversial. Analysis of whole viral genomes failed to detect evidence of A3G/F footprints ^8^, and it has been proposed that the excessive degree of G-to-A hypermutation may be lethal, even in the context of very low levels of APOBEC3G ^9^. However, later studies have suggested that whilst low-level G-to-A mutagenesis may contribute to viral evolution ^10^, the overall contribution of this effect is likely to be small ^11^.

Mutations may accumulate either because of genetic drift or because they confer a relative fitness advantage to the virus, allowing it to persist and replicate more successfully than in its previous state. Escape mutations often confer a degree of resistance against selection pressure exerted by drugs or the host immune response, and evidence of viral evolution driven by cytotoxic lymphocytes (CTLs) and antibodies can even be detected in infants ^12^. HIV-1 is known to adapt to host HLA class I ^13, 14^, and up to 56% of polymorphic sites in viral genes may be subject to HLA-associated selection pressure ^15^.

Whilst many studies have been performed *in vitro,* HIV has also been shown to evolve rapidly *in vivo* ^1^. This has obvious biological implications as untreated patients eventually lose control over the virus and progress to AIDS. Whether or not HIV continues to replicate during antiretroviral therapy (ART) is currently subject to debate, with conflicting studies suggesting evidence both for 16 and against persistent, on-going replication contributing to the maintenance of the viral reservoir during treatment ^17^.

## How does the virus evolve within and between hosts?

HIV infection is usually established from a single transmitted founder virus ^18– 20^. How this one virus then diversifies within an individual has been the subject of numerous studies. Early longitudinal studies of envelope sequences within patients identified consistent evolutionary patterns associated with disease progression ^21^ and CD4 ^+^ lymphocyte decline ^22^. In the late stages of infection, sequence divergence tends to stabilise as a result of the reduced selection pressure that can be exerted by a severely damaged immune system ^23^. As the rate of disease progression varies between patients, attempts have also been made to identify whether evolutionary factors contribute to these differences. It is now known that synonymous rather than non-synonymous substitution rates are associated with disease progression, most likely owing to underlying replication dynamics, which may in turn be driven by excessive immune activation ^24^. Primary infection may also be established from two or more strains. Such co-infections are more frequently observed with injection drug use than sexual routes of transmission, and the viruses can undergo recombination, which significantly expands the genetic diversity of the quasispecies pool ^25^.

Whilst HIV evolves extremely rapidly within individuals, viral evolution is somewhat slower on a population level, reviewed by Lemey *et al.* ^2^. Most evolutionary studies have been performed using the *env* gene, but the rate of inter-host viral evolution is consistently lower across the whole viral genome ^3^. Several hypotheses have been proposed to explain this anomaly, including fluctuations in selection pressure over time, reversion of patient-specific adaptive changes following transmission, and a “store and retrieve” mechanism in which archived ancestral virus is preferentially transmitted ^26^. The last proposal is currently the most well supported ^27^. Such cycles of latency are believed to occur irrespective of treatment, and the proportion of viruses in the plasma that have gone through latency is believed to be large ^28^. Cycling in and out of latency is therefore likely to make a non-trivial impact on viral evolution.

Another key question in HIV evolution concerns the establishment of primary infection and whether the virus responsible possesses favourable characteristics or is merely in the right place at the right time. Despite the diversification of virus within a patient, infection is typically established with CCR5 tropic (R5) virus containing specific residues associated with increased viral fitness ^29^. However, the selection bias imposed by this genetic bottleneck is significantly reduced in more permissive environments, such as in the context of inflammation, and the bottleneck itself is affected by the mode of sexual transmission ^30^. Viruses that are pre-adapted to the HLA types of the recipient are also associated with higher viral loads, rapid CD4 ^+^ lymphocyte decline, and a poorer prognosis ^31^.

## How has HIV evolved since its emergence?

HIV is an evolutionarily young virus, having first emerged in the first half of the 20 ^th^ century ^32, 33^. How the virus has disseminated and evolved since then is of great epidemiological interest, and advances in the field of phylogeography have helped to address these questions. Phylogeographic studies combine genetic and geographical data to draw inferences about historical events that have contributed to the current geographic distribution of a virus. The use of such an approach enabled the determination of the likely origin of the HIV-1 group M pandemic: 1920s Kinshasa ^34^. Group M virus is responsible for the overwhelming majority of infections worldwide, and within group M, subtype C virus is most prevalent globally ^35^.

However, it was the emergence of subtype B virus in the United States of America that first alerted medical professionals to the global AIDS crisis. A recent high-profile phylogeographic analysis of serum virus has revealed that the early US epidemic probably emerged from an existing epidemic in the Caribbean, which was introduced into New York City in around 1970 ^36^.

It has also been proposed that, since the introduction of the virus into the human population, HIV may be becoming less virulent with time. Indeed, there is some evidence to suggest that the extensive global spread of subtype C may be related to relatively lower virulence, despite comparable transmission efficiency ^37^. The rapid rate of evolution has been suggested to contribute to decreased virulence over time by allowing HIV to adapt to protective HLA types, reducing the replicative capacity of the virus ^38^. However, the interpretation of this study has been contested ^39^, and a meta-analysis of the existing literature has previously suggested the converse may be true: that the virulence of HIV is actually increasing ^40^. Further investigation is needed to clarify these apparent contradictions, which arguably have important translational implications. One possible explanation for the discrepancies between studies is that virulence may differ geographically as a result of considerable differences in the distribution of HLA class I types and HIV-1 subtypes globally.

## The evolutionary virologist’s toolkit

The number of resources available to evolutionary virologists has expanded dramatically over recent years. The latest technological advances in computing power have permitted high-level analyses of datasets to be performed, and innovative next-generation sequencing (NGS) platforms have increased—almost exponentially—the amount of data that can be collected from a sample.

Many sequencing studies continue to be performed using the conventional Sanger approach, and these investigations yield interesting and important results. The application of Bayesian Markov Chain Monte Carlo (MCMC) methods in the field of phylogenetics has revolutionised the way that sequence data are interpreted. This approach has been popularised in large part by the ability to incorporate *a priori* information about the sequences such as sampling dates. Such time-stamped data can be used to predict sequence divergence times owing to the robust molecular clock of HIV, which is itself under early immune selection pressure ^41^. Bayesian analyses also allow a large number of inferences to be drawn from sequencing data and can, for example, be used to reconstruct population dynamics ^42^ and transmission networks ^43^.

Whilst Sanger sequencing remains popular, a number of limitations are associated with traditional methods. For example, HIV sequence clustering is known to be heavily confounded by low sampling density ^44^, short sequence lengths, and suboptimal inclusion of informative sites ^45^. NGS has therefore been embraced in recent years, as it facilitates rapid, high-throughput, and cost-effective analysis of viral quasispecies. Full-length genomes can be amplified, sequenced, and assembled without bias ^46^, and the datasets generated can be of sufficient depth to allow reliable detection of ultra-rare mutations at frequencies as low as 0.2% ^47^.

Much can be learned from analysing deep sequencing data: indeed, NGS of longitudinally collected whole viral genomes from untreated patients has shown that reproducible patterns in sequence diversity between patients mirror those seen on a global scale, indicating that the fitness costs controlling diversity are universally conserved ^48^. NGS may also play an increasingly central role in clinical settings by facilitating the detection and sequencing of low-abundance virus to monitor patients for the emergence of drug-resistant variants ^49^.

NGS offers up a number of exciting opportunities for evolutionary virologists, but it is not without its own limitations. Much like Sanger sequencing, there are many possible sources of error ^50^ and, even if these are effectively overcome, the sheer size of datasets and the short fragment sizes generated may pose logistical challenges in their analysis. Progress is being made in developing novel frameworks to facilitate phylogenetic reconstruction of NGS data ^51^, but such approaches are not yet the *status quo*.

## Concluding remarks

Over recent years, a clearer picture is being formed as to how, where, and why HIV is evolving. Understanding how the virus evolves within an individual patient is central to the development of appropriate drugs and vaccines, as rapid evolution constitutes a key evasion mechanism against the immune response. Studies of viral evolution between hosts have demonstrated somewhat slower rates of evolution but have also indicated that HIV may be changing in virulence over time.

Advanced analyses and disruptive technologies have been pivotal to recent breakthroughs, and the innovative approaches being developed today will surely shape our understanding of evolutionary virology tomorrow. Particularly promising is the application of NGS to study viral evolution, as the depth and coverage of sequences generated exceed what is achievable through conventional means. In addition to being of academic interest, this technology may have important clinical implications in the future by facilitating the early detection of low-frequency drug resistance mutations and subsequently allowing alteration and optimisation of treatment plans.

To summarise, owing to a growing body of high-quality research, made possible by cutting-edge technology, HIV evolution is no longer the great enigma it once was. There are still outstanding questions to be answered, but, as more and more of these are answered, the prospect of an effective vaccine or cure becomes increasingly tangible.
